# Efficacy of garenoxacin in patients with macrolide-resistant *Mycoplasma pneumoniae* pneumonia

**DOI:** 10.1128/aac.00025-26

**Published:** 2026-06-11

**Authors:** Naoyuki Miyashita, Makoto Ogata, Naoki Fukuda, Akihisa Yamura, Tomoki Ito

**Affiliations:** 1 First Department of Internal Medicine, Division of Respiratory Medicine, Infectious Disease and Allergology, Kansai Medical University 12880, Hirakata, Japan; University of Fribourg, Fribourg, Switzerland

**Keywords:** garenoxacin, macrolide-resistance, macrolide-sensitive, minocycline, *Mycoplasma pneumoniae*

## Abstract

The prevalence of macrolide-resistant *Mycoplasma pneumoniae* in Japan is increasing once again. The therapeutic efficacy of quinolone garenoxacin was compared with minocycline, which is recommended as the first-choice drug in patients with macrolide-resistant *M. pneumoniae* pneumonia. Defervescence within 48 h after initiation of antibiotics was observed in 90.6% of garenoxacin and 90% of minocycline recipients. Our results indicated that garenoxacin can be considered as the first-choice drug for the treatment of macrolide-resistant *M. pneumoniae* pneumonia.

## INTRODUCTION

Garenoxacin (Toyama Chemical Co., Ltd.) is a novel des-F(6)-quinolone antibacterial drug that lacks a fluorine substituent at the C-6 position in the quinolone ring, which had been believed to be indispensable in past fluoroquinolone antibacterial drugs (1). Garenoxacin has demonstrated highly potent activity against major respiratory pathogens (2–5), and clinical studies have confirmed its efficacy against atypical and bacterial pneumonia (6, 7). Furthermore, among quinolones, garenoxacin is less susceptible to drug resistance (8–12). For this reason, the Japanese Respiratory Society (JRS) pneumonia management guidelines, revised in 2024, recommended garenoxacin as the first-line drug for the empirical treatment of community-acquired pneumonia (CAP) (13).

Macrolide-resistant*Mycoplasma pneumoniae* emerged in Japan in 2000 and has since spread rapidly, primarily in East Asia (14–16). In 2012, the proportion of macrolide-resistant *M. pneumoniae* reached 80–90%. Since then, resistant strains have decreased year by year to 20–30% (17). However, since 2019, macrolide-resistant *M. pneumoniae* has begun to increase again, reaching 60% in 2024, raising concerns about further spread (17). While the efficacy of garenoxacin has been confirmed for macrolide-sensitive *M. pneumoniae* (6), no studies have examined the efficacy of garenoxacin for macrolide-resistant *M. pneumoniae*. In this study, we compared the efficacy of garenoxacin with minocycline for macrolide-resistant and -sensitive *M. pneumoniae* pneumonia to verify the validity of the revised JRS pneumonia guidelines. Minocycline was selected as the comparator drug because it is recommended as the first-line drug for *M. pneumoniae* pneumonia in the JRS pneumonia guidelines (13).

This study was a non-randomized, physician-led antibiotic selection trial conducted at 12 facilities affiliated with Kansai Medical University Hospital between January 2024 and December 2025. Patients were selected from those with community-acquired pneumonia who presented to the hospital for the first time before antibiotic administration. A rapid Mycoplasma antigen test was performed on patients with a Mycoplasma Pneumonia Diagnostic Prediction Score of 5 or higher (specificity 99%), as recommended in the JRS pneumonia guidelines. Patients with positive antigen results were registered, and antibiotics were initiated before the resistance genes were identified. Cultivation and polymerase chain reaction (PCR) testing for *M. pneumoniae* were carried out as previously reported (16–18). All antigen-positive cases were also PCR-positive.

A direct sequencing method was used to search for mutations at sites 2063, 2064, and 2617 in the *M. pneumoniae* 23S rRNA domain V gene region in isolates or samples with a positive PCR result, as previously reported (16–18). The severity of pneumonia was evaluated using the A-DROP system of predictive rules proposed by the JRS pneumonia guidelines (13). Frequencies were compared using Fisher’s exact test. Between-group comparisons of normally distributed data were performed using a Student’s *t*-test. Skewed data were compared using the Mann-Whitney*U* test.

The therapeutic efficacy of garenoxacin and minocycline against macrolide-sensitive and -resistant *M. pneumoniae* pneumonia was evaluated. The attending physicians made the antibiotic selection. Garenoxacin was administered at a dosage of 400 mg once daily and minocycline at 200 mg twice daily in accordance with the package insert accompanying each drug. If clinical symptoms and signs had not improved by the second visit, the attending physicians changed the antibiotic treatment to minocycline or other quinolones (examples include lasufloxacin, levofloxacin, tosufloxacin, moxifloxacin, and sitafloxacin).

We assessed the clinical outcome using fever, as previous studies have also only used the number of days with a fever to evaluate the clinical outcome in macrolide-resistant patients (16–20). Additionally, we evaluated the therapeutic efficacy of antibiotic treatment after 48 h, as previous reports have shown that the mean number of days with a fever in macrolide-sensitive patients during macrolide administration is between 1 and 2 days (19, 20). The number of days with fever was defined as the day antibiotics were started, and fever reduction was defined as an initial body temperature of less than 37.0°C persisting for 24 h.

During the study period, 71 cases of *M. pneumoniae* pneumonia were enrolled, of which 45 were macrolide-resistant. All of these patients had an A-to-G transition at position 2063 in domain V of the 23S rRNA gene (A2063G). Table 1 shows the patient background and clinical findings in the garenoxacin- and minocycline-treated groups. All patients had a fever of 38°C or higher at the start of treatment. There were no significant differences in clinical features between the two groups.

**TABLE 1:** Underlying conditions and clinical findings in patients with *Mycoplasma pneumoniae* pneumonia at the first examination between the garenoxacin and minocycline groups^*b*^

Variables	Garenoxacin group	Minocycline group	*P* value
No. of patients	51	20	
Median age (IQR), years	21.5 (17–30)	20.8 (16–29)	0.6776
No. of males/females	24/27	9/11	>0.9999
No. (%) of patients with co-morbid illnesses	4 (7.8)	2 (10.0)	>0.9999
No. (%) of patients with each pneumonia severity score^*a*^			
Mild	40 (78.4)	16 (80.0)	>0.9999
Moderate	11 (21.6)	4 (20.0)	>0.9999
No. (%) of patients with macrolide-resistant *Mycoplasma pneumoniae* pneumonia	32 (62.7)	10 (50.0)	0.4224
No. (%) of patients with the following clinical signs and symptoms			
Fever (≥38.0°C)	51 (100)	20 (100)	>0.9999
Cough	50 (98.0)	19 (95.0)	0.4869
Sputum production	31 (60.8)	13 (65.0)	0.7922
Sore throat	20 (39.2)	9 (45.0)	0.7894
Headache	16 (31.4)	5 (25.0)	0.7742
Shortness of breath	4 (7.8)	2 (10.0)	>0.9999
Laboratory findings, median (IQR)			
White blood cells, mean (/µL)	6,400 (5,300–8,700)	6,600 (5,500–8,800)	0.6288
C-reactive protein, mean (mg/dL)	4.6 (1.8–9.5)	3.5 (1.1–8.5)	0.5984
Aspartate aminotransferase, U/L	26 (20–37)	27 (21–38)	0.7351
Alanine aminotransferase, U/L	24 (19–36)	25 (18–37)	0.7426

^*a*^ Patients were stratified into four severity classes by the A-DROP system: 0 point = mildand 1 or 2 points.

^*b*^ Continuous values are presented as medians and interquartile ranges and categorical/binary values as counts and percentages.

Table 2 shows the treatment outcomes between the two groups. For patients with macrolide-sensitive *M. pneumoniae* pneumonia, fever subsided within 48 h in 94.7% of patients in the garenoxacin group and 90% of patients in the minocycline group. For patients with macrolide-resistant *M. pneumoniae* pneumonia, fever subsided within 48 h in 90.6% of patients in the garenoxacin group and 90% of patients in the minocycline group. In both groups, there were no cases requiring a change in antibiotic or the use of additional antibiotics, and all patients were cured.

**TABLE 2:** Clinical efficacies of garenoxacin and oral minocycline against mild to moderate macrolide-sensitive and macrolide-resistant *M. pneumoniae* pneumonia

	Macrolide-sensitive patients:		Macrolide-resistant patients:	
Variable	Garenoxacin	Minocycline	Garenoxacin	Minocycline
No. of patients	19	10	32	10
Mean age, years	22.1	21.8	21.3	20.5
No. of males/females	9/10	4/6	15/17	5/5
No. (%) of patients with defervescence within 48 h after antibiotic administration	18 (94.7)	9 (90.0)	29 (90.6)	9 (90.0)
No. (%) of patients with antibiotic change at second visit	0	0	0	0

The JRS pneumonia guidelines revised in 2024 recommend garenoxacin, which is less likely to induce resistance, as the first-line drug for empirical treatment of CAP (13). Garenoxacin is effective against major bacterial pathogens that exhibit drug resistance (13, 21), but its efficacy against drug-resistant *M. pneumoniae* strains had not been evaluated. Our current study confirmed that the efficacy of garenoxacin against macrolide-resistant *M. pneumoniae* pneumonia is equivalent to that of minocycline, which is the first-line drug for macrolide-resistant *M. pneumoniae* pneumonia (13, 21). To date, no *M. pneumoniae* strains resistant to fluoroquinolones have been reported anywhere in the world.

The limitations of this study include a non-randomized trial design, physician-driven antibiotic selection, and the use of fever resolution as the only clinical outcome without considering microbiological or radiological endpoints. Furthermore, there was an imbalance between the number of patients treated with garenoxacin and minocycline, and this discrepancy may affect the statistical significance of the results. Further investigation is needed.

In conclusion, our results demonstrated that garenoxacin may be effective as a treatment for macrolide-resistant*M*. *pneumoniae* pneumonia. If minocycline cannot be used, garenoxacin should be considered.

## Data Availability

All the data presented in this study are available in the article.
